# Characterization of Key Aroma Compounds in Dongpo Pork Dish and Their Dynamic Changes During Storage

**DOI:** 10.3390/foods14071084

**Published:** 2025-03-21

**Authors:** Min Xu, Yang Kang, Ying Wang, Lan Li, Yu Liu, Qin Xiang, Hongbin Lin, Ping Liu, Jie Tang

**Affiliations:** 1 College of Food and Biotechnology, Xihua University, No. 999 Guangchang Road, Chengdu 610039, China; minxu_sein@163.com (M.X.); 13378117702@163.com (Y.K.); ying_wang555@163.com (Y.W.); 18880403225@163.com (L.L.); 13550215271@163.com (Y.L.); xiangqin@xhu.edu.cn (Q.X.); hongbin-ok@163.com (H.L.); tangjie1225@mail.xhu.edu.cn (J.T.); 2 China Agricultural University-Sichuan Advanced Agricultural & Industrial Institute, Chengdu 611430, China; 3 Chengdu Guo Niang Food Co., Ltd., Chengdu 610500, China

**Keywords:** Dongpo pork dish, aroma characteristics, volatile profiles, sensomics-based identification, storage stability

## Abstract

The objective of this study was to identify the key aroma compounds of Dongpo pork dish (DPD) and to explore the changes in key aroma compounds of DPD during the storage period. Quantitative descriptive analysis (QDA) combined with two-dimensional gas chromatography-mass spectrometry (GC×GC-MS) was employed to investigate the aroma characteristics and the volatile profiles of DPD. Further, a sensomic approach was used to decipher its key aroma compounds. The typical flavors identified in DPD were described as meat, grease, garlic, wine, soy sauce, and spice flavors by the QDA. The key aroma compounds contributing to the flavor of DPD include 2-heptanol, 1-octen-3-ol, hexanal, (E)-2-octenal, 3-methylthiopropanal, decanal, ethyl caproate, 2,5-dimethylpyrazine, and dimethyl trisulfide. In addition, the changes of key aroma compounds of DPD at different storage temperatures (25 °C, 4 °C) were explored, and the results demonstrated that the key aroma compounds showed an overall trend of attenuation with the increase in time. The content of ethyl caproate decayed by more than 60%. Compared with the storage temperature of 25 °C, DPD storage at 4 °C was more effective in slowing down the change of key aroma compounds. These results can provide theoretical evidence for the flavor modulation and the industrial production of DPD.

## 1. Introduction

With the rapid development of the economy, consumer demand for dietary nutrition, efficiency, and convenience is becoming remarkably urgent. Therefore, the prepared dish, known for being convenient, has witnessed a spurt of growth. The prepared dish mainly refers to the production of traditional food with industrial technology, which has gained a lot of attention. Dongpo pork dish (DPD) is a traditional characteristic dish of China with great popularity at home and abroad. The prepared production of DPD makes it more accessible to consumers. In order to improve the quality of prepared DPD products, a deep investigation of its flavor is of great importance. The characteristic flavor of DPD mainly forms through two key stages: frying and steaming, with some specific condiments. Hence, the flavor quality of DPD is tightly correlated with the quantities and varieties of spices used during processing, as well as the technological parameters like producing temperature and time. However, the spices and some of the production technological parameters are not unambiguously specified and usually vary in different companies for the industrialized production of the traditional DPD, which leads to the uneven flavor characteristic of the industrialized DPD products in the market. As for consumers, it is hard to recognize the “real flavor” of DPD. Therefore, in order to reveal the characteristic flavor of DPD, it is necessary to systematically analyze the DPD products from different sources to elucidate their similarity, and in a further step, provide theoretical support for the production of DPD with standardized flavor.

Meanwhile, the flavor of DPD products during storage and circulation decay, resulting in the differences between the authentic flavor of DPD and the industrialized products, which restricts the further quality development of the industrialized DPD products. Therefore, there is an urgent need to further clarify the change pattern of characteristic aroma compounds during storage, and ultimately to provide a theoretical basis for the quality improvement of industrialized DPD.

The study of food flavor has attracted extensive attention from researchers. Flavor, including aroma and taste, is one of the most important sensory quality characteristics of meat-prepared dishes, and are important attributes that influence consumer acceptability of meat products [1,2]. At present, gas chromatography-mass spectrometry (GC-MS), two-dimensional gas chromatography-mass spectrometry (GC×GC-MS), odor activity value (OAV), the gas chromatography-olfactometry-mass spectrometry (GC-O-MS), electronic nose (E-nose), quantitative descriptive analysis (QDA), and aroma recombination and omission tests have been applied to the analysis of active compounds [3,4,5]. GC-MS is capable of separating and identifying food samples [6]. However, the complexity of food components leads to long sample preparation and processing times [7]. In 1991, Phillips first proposed GC×GC, which can overcome limitations in the separation process of complex samples by synergistically separating two columns with different stationary phases [8]. Compared with GC, GC×GC effectively improved the separation and identification accuracy of volatile compounds in foods and thus has been applied to analyze volatile compounds in various food products [9]. Li et al. [10] used E-nose combined with GC×GC-MS to investigate the aroma changes of DPD throughout the production process. Zhao et al. [11] used GC×GC-MS to detect volatile compounds from different parts of grass carp. However, a comparison of the fragments of compounds detected by GC×GC-MS with the standard reference fragments in the database could not determine the odor characteristics of the compounds and their contributions to the sample. GC-O-MS can combine the sensitivity of the human nose with the qualitative technique of MS, and it is able to determine the odor contribution of aroma compounds. OAV is an important index for judging aroma intensity, and the aroma recombination and omission tests, which are based on OAV ≥ 1, describe and reconstruct the flavor profiles in food with the smallest number of compounds and the most accurate content, and identify the key aroma compounds. In the study of Zhao et al., 54 volatile compounds were identified by GC-MS in ginger stewed beef, and then 23 compounds were detected by GC-O and further quantified, in which the OAV of 20 aroma-active compounds was higher than 1 [12]. Finally, 19 key aroma compounds were identified by using aroma recombination in combination with omission tests. The combined application of these advanced techniques could comprehensively reveal the characteristic volatile profile of foods, which serves as a theoretical foundation for systematic research on the flavor characterization of DPD.

Therefore, QDA and GC×GC-MS were firstly combined to analyze the aroma characteristics and the volatile profiles of DPD in this study. The contribution of volatile compounds to the overall aroma of DPD was determined by the OAV and GC-O-MS. In a further step, the aroma recombination and omission experiments were introduced to verify the key aroma compounds. Meanwhile, in order to reveal the changes in the main volatile profile of DPD during storage, the key aroma compounds were further investigated by using GC×GC-MS in different storage conditions. This research aims to provide a basis for the subsequent flavor modulation and the construction of the DPD flavor standard.

## 2. Materials and Methods

### 2.1. Sample Preparation

As shown in Table 1, samples B, C, and D were obtained from different restaurants in Chengdu, and Sample E was a purchased pre-prepared dish. Sample A was prepared according to the local standard “Technical Specification for Sichuan Cuisine Dongpo Meat Cooking Process (DB51/T 2435-2017) [13]. Fresh pork (800 g) was cut into cubes (4 × 4 × 4 cm), and the following ingredients were added: ground spices (20 g), minced garlic (200 g), pickled peppers (200 g), salt (8 g), soy sauce (20 g), dark soy sauce (20 g), single crystal rock sugar (28 g), white wine (200 mL), monosodium glutamate (20 g), chicken seasoning (20 g) and black beans (20 g), then the mixture was simmered for 60 min. All the seasonings used were purchased from the local Walmart Supermarket (Chengdu, China). All samples were stored at −18 °C.

In this study, most of the chemicals and reagents were from Macklin Biochemical Technology Co., Ltd. (Shanghai, China): lauraldehyde (95%), δ-decanolactone (97%), 4-hydroxy-2,5-dimethyl-3(2H)furanone (98%), 4-Hydroxy-3-methoxystyrene (98%), 2,3-dimethylpyrazine (98%), 4-carvomenthenol (98%), 2,3-pentanedione (98%), decanal (97%), (E)-2-octenal (95%), methional (98%), 2,3,5-trimethylpyrazine (99%), dimethyl trisulfide (98%), 5-methyl-2-hexanone (99%), 2-heptanol (99.5%), (E)-2-octen-1-ol (95%), 1-octen-3-ol (98%), furfuryl alcohol (98%), ethyl hexanoate (99%), nonanal (96%), benzaldehyd (99.5%), 4-allyl anisole (97%), caproaldehyde (99%), eucalyptol (99%). Terpene olefin (95%) and 2,6,6-trimethyl-2,4-cycloheptadien-1-one (98%) were obtained from Yuanye Biotechnology Co., Ltd. (Shanghai, China), and 2-methyl-3-heptanone (99%) as internal standard and n-alkane (C_8_–C_25_, 97%) were obtained from Sigma-Aldrich Co., Ltd. (Shanghai, China). Chromatographically grade methanol was purchased from Cologne Chemical Co., Ltd. (Chengdu, China).

### 2.2. Quantitative Descriptive Sensory Analysis

The quantitative descriptive sensory analysis was conducted according to the method of Wang et al. with some modifications [14]. Ten members (five females and five males, aged 23–28 years) with extensive experience in descriptive analysis were recruited by evaluating basic tastes, olfactory matching, paired tests, ranking tests, and descriptive skills according to the international standards (ISO 6658:2017-07) [15]. All panelists were trained on different samples to ensure consistency and reproducibility of the assessment. Panelists were asked to give descriptive terms for the recipe. Six aroma terms and overall flavor were selected for descriptive analysis (Table 2). Panelists were instructed to assess the samples (5 g) by using a ten-point interval scale (0 = not present and 9 = extremely strong). All samples were provided to the evaluator at the same time and were randomly coded. Each sample was performed in triplicate. The purified water was provided to clean the mouth in between evaluations of different samples. The obtained seven-dimensional aroma wheel was plotted in a spider web diagram. All sensory experimental procedures were approved by the Ethics Committee of Xihua University, China.

### 2.3. GC×GC-MS Analysis

Headspace solid-phase microextraction (HS-SPME) was referred to as the method of Zhong et al. with slight modifications [16]. A 3.00 g sample was stirred, and then the sample was poured into a headspace vial with 2-methyl-3-heptanone (1 μL 0.816 mg/mL) as the internal standard. After equilibrating in a water bath at 80 °C for 10 min, the extractor head (75 μm CAR/PDMS) was inserted into the headspace vial for 45 min of extraction and then desorbed for 3 min in a GC injection port.

The GC×GC-MS (GC 2010, Shimadzu, Japan) method referenced the description of Li et al. [10] with some modifications. The first-dimensional column was DB-Wax (30 m × 0.25 mm × 0.25 μm, Agilent Technologies, Santa Clara, CA, USA), the second-dimensional column was DB-17 MS capillary column (1.2 m × 0.18 mm × 0.18 μm, Agilent Technologies, Santa Clara, CA), modulation column was HV series modulation column (C_8_–C_25_). The carrier gas was high-purity helium (purity ≥ 99.999%) at 1.0 mL/min. The inlet temperature was 250 °C. The programmed temperature increase: the starting temperature was 40 °C, held for 2 min, and then the temperature increased to 240 °C at 6 °C/min. The temperature of the ion source was 230 °C, the interface temperature was 250 °C, and the mass scan range of *m*/*z* was set from 41 to 330. Internal standard 2-methyl-3-heptanone (99%) and external standard n-alkane (C_7_–C_30_, 97%) were used for qualitative and quantitative analysis. The laboratory uses Canvas 2.0 for data processing.

### 2.4. OAV Analysis

The OAV was determined by calculating the ratio of the content of each aroma component to the threshold value, and the contribution of the aroma component to the overall flavor of the sample was evaluated by the following formula:

(1) OAV=Ci⁄Ti

where C*i* was the concentration of volatile flavor compound, mg/kg. T*i* was the sensory threshold value of the corresponding volatile flavor compound in oil, mg/kg [17].

### 2.5. GC-O-MS Analysis

A GC-O-MS analysis was carried out via GC-MS with a Gerstel’s ODP-3 sniffer port for the detection of aromatic compounds and the pretreatment method and instrument setup were consistent with GC×GC-MS. The aroma assessment method referred to the method of Ke et al. [18] with some modifications. An evaluation team of three evaluators with experienced sensory judgment and special training was formed with a view to assessing the aroma compounds using GC-O-MS. The evaluators performed independent sniffing and recorded the time of occurrence, character, and aroma intensity (AI). The AI of the aroma compounds was recorded on a scale of 0–5 (0 = not present, 3 = moderate, and 5 = strong), and each test was repeated three times.

### 2.6. Aroma Recombination and Omission Tests

Recombination model (Model 1): The recombination model was one in which the key volatile flavor standards from the identification analysis were added to the flavorless matrix at the detected concentrations. The recombination model was prepared using an odorless matrix, which was prepared from glycerol octanedecanoate and equivalent aroma compound standards.

Omission model (Model 2): Omission tests were carried out by omitting single off-odorants including 1-pentanol, 2-heptanol, 1-octen-3-ol, hexanal, n-octanal, nonanal, (E)-2-octenal, 3-methylthiopropanal, benzaldehyde, decanal, (Z)-2-nonenal, ethyl caproate, 2-n-pentylfuran, 2,5-dimethylpyrazine, dimethyl trisulfide, and diallyl disulfide. Ten members (five females and five males, aged 23–28 years) with extensive experience in descriptive analysis were also recruited to evaluate the aroma attributes of a series of omission models. The triangle test was adopted for the evaluation of each omission model [19].

### 2.7. Storage Experiments of DPD

In the sample preparation stage, all samples were stored at −18 °C and sealed in plastic bags. In the test phase, the samples were stored at 25 °C and 4 °C for 30 days, respectively. The samples were taken every 5 days. Then GC×GC-MS was used to detect the volatile organic compounds (VOCs).

### 2.8. Statistical Analysis

Data processing was performed using SPSS Statistics 26.0 software for data analysis and the DUNCAN test in one-way analysis of variance (one-way ANOVA) for significance analysis (*p* < 0.05), and all experimental results were expressed as means ± standard deviations. A correlation analysis was conducted using Origin Pro 2022b software (Origin Lab Corporation, Northampton, MA, USA), principal component analysis was conducted using SIMCA 14.1 software, and the heatmap plotting was performed using Tbtools (2.0 version).

## 3. Results and Discussion

### 3.1. Aroma Characteristics of DPD from Different Companies

**Figure 1:**
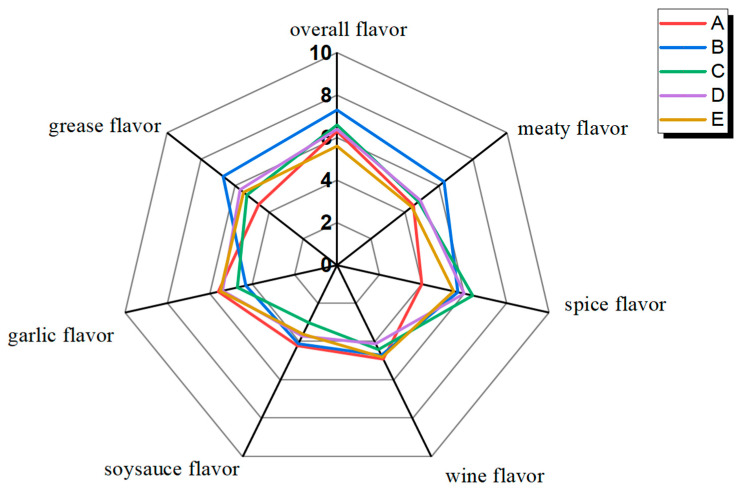
Quantitative descriptive sensory analysis of DPD.

In the series of characteristic sensory descriptors of DPD, the sensory evaluator screened out six characteristic sensory descriptors of DPD. ANOVA was employed to distinguish differences between different samples based on the six descriptors. As shown in Figure 1, the typical aromas of DPD were described as Meat flavor, Grease flavor, Garlic flavor, Wine flavor, Soy Sauce flavor, and Spice flavor. The sensory scores of garlicky, meaty, and greasy were higher than the other attributes. Notably, sample B had the highest overall flavor score and higher scores for meaty and greasy (*p* < 0.01), indicating that meaty and greasy might contribute to the overall flavor. Meaty and greasy were two important indicators of the overall aroma quality of pork products [14]. Meaty and greasy usually come from the oxidation and degradation of animal lipids and the Maillard reaction between amino acids and sugars, with most of its key aroma compounds being aliphatic aldehydes, ketones, and alcohols [20,21]. Sample D had the lowest score (*p* < 0.01) for the wine flavor, which might be due to the loss of volatile flavor compounds that presented wine flavor during storage.

### 3.2. Volatile Compound Profiles of DPD Based on GC×GC-MS

**Figure 2:**
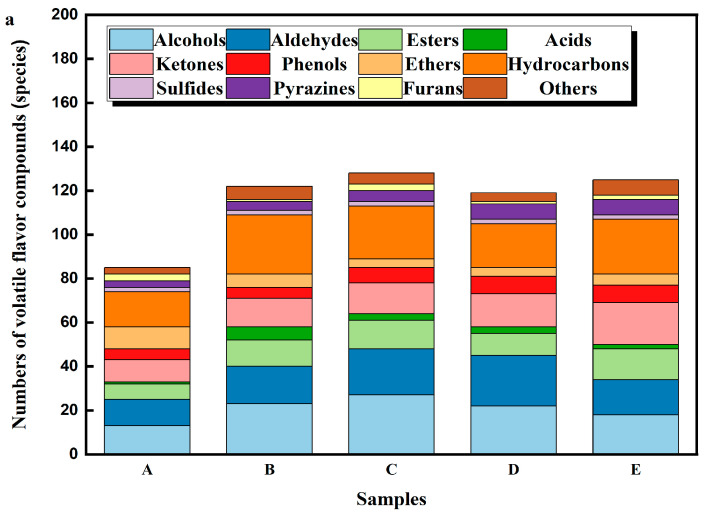

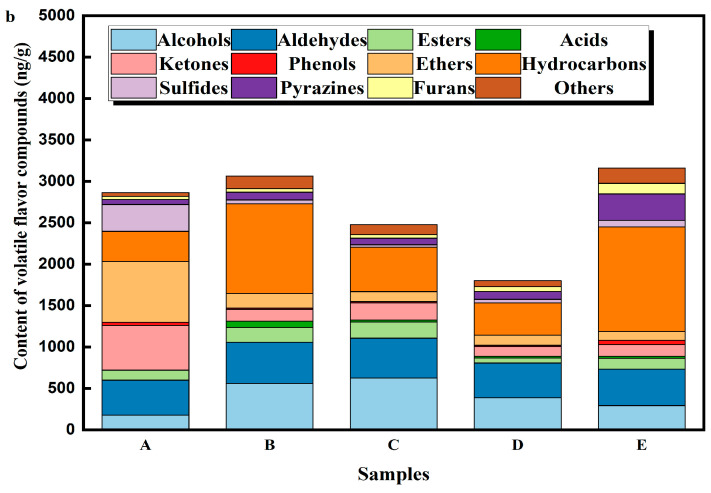
The number of volatile flavor compounds in DPD from different sources (**a**) and the content of volatile flavor compounds in DPD from different sources (**b**).

As shown in Figure 2a, 85, 122, 128, 119, and 125 VOCs were, respectively, identified in samples A, B, C, D, and E, mainly including 41 alcohols, 32 aldehydes, 8 acids, 28 esters, 34 ketones, 11 ethers, 16 phenols, 44 hydrocarbons, 8 pyrazines, 5 furans, and 16 other compounds. The total contents of samples A, B, C, D, and E were 2864.33 mg/kg, 3064.56 mg/kg, 2476.89 mg/kg, 1800.77 mg/kg, and 3158.29 mg/kg, respectively, and hydrocarbons and alcohols were the most abundant. Figure 2b shows the content of different types of VOCs, while the specific concentrations of each volatile compound can be seen in Supplementary Materials.

Alcohols were formed mainly via sugar metabolism, lipid oxidation, and decarboxylation and dehydrogenation of amino acids, and had a soft flavor [22,23]. A total of 41 alcohols (2047.05 mg/kg) were detected in the five DPD samples, which played an important role in the overall aroma of the samples. In addition, 1-pentanol, 2-heptanol, and 1-octen-3-ol were detected in all samples. 1-Pentanol might derive from the oxidation of linoleic acid in meat during cooking and has a fruity and oily flavor [24]. 1-Octen-3-ol might derive from the β-oxidation of long-chain polyunsaturated fatty acids, which gave meat products a mushroom flavor [25,26]. As for 2-heptanol, it was usually found in tea and ginger and mainly presented sweet and floral-fruity flavors [27]. In this experiment, it might originate from the spice powder.

In meat products, aldehydes were generated mainly by lipid oxidation or Strecker degradation. Due to their high concentration (2257.29 mg/kg) and low threshold, aldehydes were important flavor contributors for meat products. A total of 32 aldehydes were detected in this experiment, mainly including hexanal, octanal, nonanal, (E)-2-octenal, 3-methylthiopropanal, decanal, and (Z)-2-nonenal. Hexanal and decanal mainly presented grassy and vegetable flavors, which came from the oxidation of unsaturated fatty acids. Octanal and nonanal presented fresh and sweet aroma [28]. Benzaldehyde might derive from the decomposition of phenylalanine or linoleic acid and presents a bitter almond flavor [29]. 3-methylthiopropanal, (E)-2-octenal, (Z)-2-nonenal, and decanal had a significant influence on the flavor of fats [30,31].

Ketones might derive from β oxidation of saturated fatty acids, amino acid degradation, Maillard reaction, and esterification with microorganisms, and were the result of further oxidation of aldehydes. Ketones had a great influence on the flavor of meat products as they had specific flavors. It was noteworthy that 4-octanone, which was present in sample A at a high level of 426.88 mg/kg, was associated with the creamy and fatty flavor of meat products [32]. A total of 34 ketones (1138.62 mg/kg) were detected, and 3-hydroxy-2-butanone was detected in all the samples, which might originate from glucose catabolism or oxidation of oleic acid and had the odor characteristics of fat [33].

Esters were formed by esterification between alcohol and fat. In the present study, 28 esters were detected with a total content of 682.93 mg/kg. Among all the samples, sample A had the highest content of ethyl caproate (67.39 mg/kg), which mainly presented wine and fruity flavor. In the study of Bi et al., the significant increases in the number of key aroma compounds, such as ethyl caproate, with an increased cooking time resulted in a more meat flavor [34].

Pyrazines and furans were common results of the reaction of free amino acids, peptides, and proteins under heat treatment [35]. In the present study, there were eight pyrazines and five furans, which accounted for 7.2% of the total content. Pyrazines provided nutty and barbecue flavors to meat products. It was reported that 2,5-dimethylpyrazine could provide cocoa and coffee flavors [36]. Furans might derive from Maillard’s reaction. 2-pentylfuran, a non-carboxylic compound derived from linoleic acid and other n-6 fatty acids, presented a nutty and vegetable flavor [37].

In the present study, a total of 16 phenols, 8 acids11, ethers, 44 hydrocarbons, 2 sulfides, and 16 other compounds were detected. Acid volatile compounds might be formed by the conversion of aldehydes to the corresponding alcohols by alcohol dehydrogenases and then by aldehyde dehydrogenases, while hydrocarbons as well as other compounds were mainly derived from long-chain fatty acids or spices [38,39]. In all samples, higher contents of 3-carene, β-pinene, terpinolene, 1-stigmasterene, dimethyl trisulfide, and allyl methyl disulfide were detected, which were mainly associated with spices. For example, allyl methyl disulfide and dimethyl trisulfide were both major volatiles in garlic [40]. 3-carene, β-pinene, terpinolene, and 1-stigmasterolene were present in spices such as cloves, cinnamon leaves, star anise, cardamom, and nutmeg fruits [41,42,43].

### 3.3. Analysis of Shared VOCs of DPD from Different Companies

**Figure 3:**
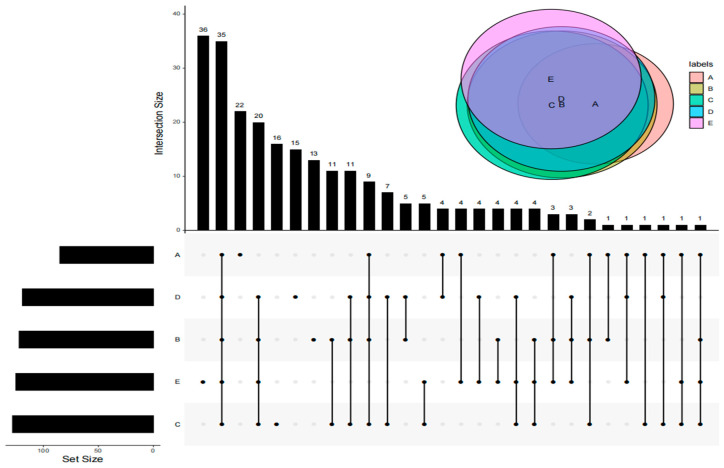
Advanced Wayne diagram of all volatile compounds in DPD from different sources.

**Figure 4:**
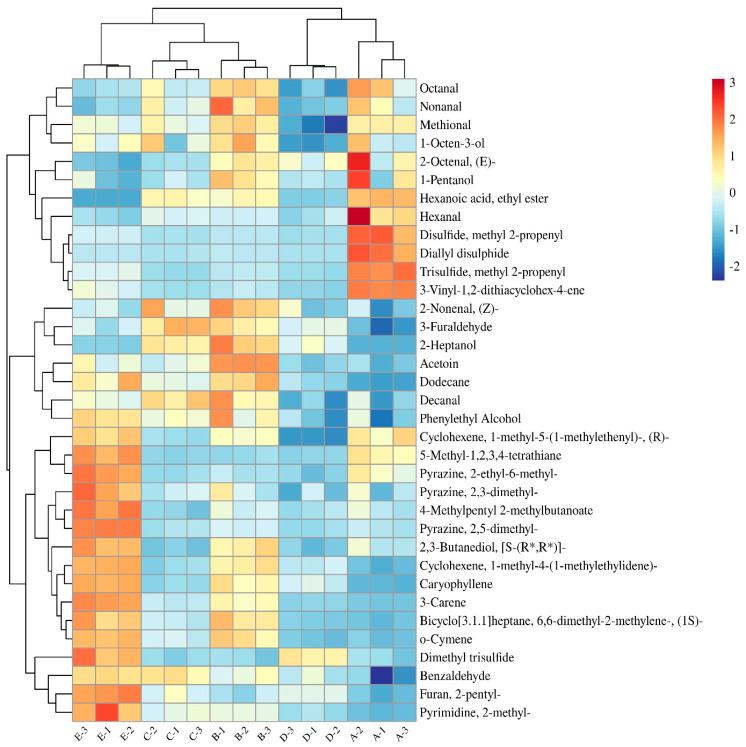
Heatmap of shared VOCs in DPD.

The advanced Wayne diagram can clearly distinguish the common volatiles of the five DPD samples. As shown in Figure 3, there were a total of 35 shared compounds in the five DPD samples, including nine aldehydes, five alcohols, two ethers, one ketone, one ester, eight hydrocarbons, one furan, three pyrazines, four sulfides, and one other compound. The results showed that samples A and E were clearly distinguished from other samples in terms of volatile compounds. Heatmap analysis of these 35 shared compounds showed that the five samples were mainly classified into three clusters, as shown in Figure 4. Cluster 1 included samples A and D, cluster 2 included samples B and C, and cluster 3 included sample E. Aldehydes had the largest number of species among all shared compounds and were the key volatile compounds. Compared with the other samples, the content of (E)-2-octenal, 1-pentanol, ethyl hexanoate, hexanal, 3-vinyl-1,2-dithio-4-cyclohexene, methyl-2-propenyl-disulphide, and methyl-2-propenyl-trisulphide were higher in sample A. These compounds contributed to the fat, wine, and garlic flavors of DPD. It was also noteworthy that the contents of olefins and pyrazines were higher in sample E, and olefins were mainly associated with spices. It was reported that stigmasterol was a natural compound present in plants such as bay leaves, basil leaves, and cloves, eliciting a mild flavor of woody and fruity notes [44].

### 3.4. Identification of Aroma-Active Compounds Using OAV and GC-O-MS Analysis

The Odor Activity Value (OAV) allowed for a reasonable assessment of aroma contribution. The VOCs with OAV ≥ 1 were usually considered aroma-active compounds, contributing a lot to the overall aroma [45]. Combined with GC-O-MS, the AI values of the shared compounds were obtained based on the acute smell sense of human beings through the time-intensity method, and usually, the VOCs with AI ≥ 2 contributed significantly to the overall aroma. As shown in Table 3, the OAV of five aldehydes, two alcohols, one ester, one furan, and one sulfide were over one, among which the OAV of 3-methylthiopropanal, 1-octen-3-ol, and dimethyl trisulfide were greater than or equal to 100, indicating that these three compounds were more important for the “meat aroma” and “garlic aroma” of DPD. A total of 35 shared aroma active compounds were perceived by GC-O-MS, in which the aroma compounds with AI ≥ 2 included seven aldehydes, three alcohols, one ketone, two alkanes, six olefins, three pyrazines, one furan, and four other compounds, among which 3-methylthiopropanal, 1-octen-3-ol, and dimethyl trisulfide had higher AI (AI ≥ 3). This was consistent with the OAV results. Yang et al. [46] also used OAV to identify key aroma compounds in dry-refined beef fat, including 2-methyl-3-furanethiol, 3-methylthiopropanal, (E, E)-2,4-nonadienal, 12-methyltridecanal, and 1-octen-3-one. Ultimately, 16 VOCs with AI ≥ 2 or OAV ≥ 1 were identified as aroma active compounds, including 1-pentanol, 2-heptanol, 1-octen-3-ol, hexanal, n-octanal, nonanal, (E)-2-octenal, 3-methylthiopropanal, benzaldehyde, decanal, (Z)-2-nonenal, ethyl caproate, 2-n-pentylfuran, 2,5-dimethylpyrazine, dimethyl trisulfide, and diallyl disulfide.

### 3.5. Identification of Key Aroma Compounds Using Aroma Recombination and Omission Tests

To better determine the contributions of the aroma-active compounds, the 16 aroma-active compounds were quantitatively calibrated using standard curves (Table 4). After calibration, their contributions to the overall aroma of DPD were further verified through aroma recombination and omission tests.

**Figure 5:**
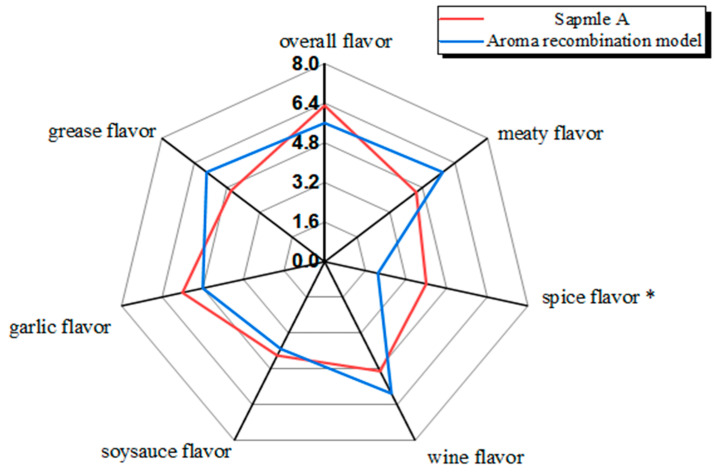
Sensory radar diagrams of the recombination model of DPD and the original. sample A. * indicated a significant difference in spice flavor.

As shown in Figure 5, there were slight differences between the recombination model and the original sample in volatile profile. This may be related to the masking, inhibition or synergistic effect between the compounds with OAV < 1 and the compounds with OAV > 1. Statistical analysis of the data showed that the recombination model scored 5.6 points for “overall flavor”, and there was no significant difference between the aroma recombination model and DPD (*p* > 0.05). On the other hand, there was a significant difference in “spice flavor”, which was due to the composition of spice flavor being complex and it cannot be completely reproduced by several simple flavor specimens. The results of the recombination tests confirmed the results of the identification and quantification tests. Further aroma omission tests were used to investigate the contributions of specific compounds or a group of compounds to the overall odor of DPD. According to Table 5, a total of 16 omission models were prepared, and then omission models were compared with the recombination model containing all compounds with AI ≥ 2 or OAV ≥ 1. The results showed that the absence of 2-heptanol and 1-octen-3-ol could lead to a significant difference (*p* ≤ 0.01 and *p* ≤ 0.001, respectively). It was implied that the clear, mushroom, and floral alcohols contributed significantly to the aroma of DPD, and the omission model with the absence of 1-pentanol was found to be associated with low fruity flavor, but there was no significant difference (*p* > 0.05). When omitting hexanal-free, (E)-2-octenal, and 3-methylthiopropanal, panelists could perceive a high significance (*p* ≤ 0.01) between each omission model and recombination model, but there were no significant differences (*p* > 0.05) between the four omission models of octanal, nonanal, benzaldehyde, and (Z)-2-nonenal. In addition, 2,5-dimethylpyrazine and dimethyl trisulfide omission models also had significant differences (*p* ≤ 0.05 and *p* ≤ 0.01, respectively). 2-pentylfuran was usually considered to be an oxidation product of ω-6 polyunsaturated fatty acids, which had a potato aroma [47]. However, due to its low content and high odor threshold, it was not identified as an important flavor compound in the present omission model. The results showed that 2-heptanol, 1-octen-3-ol, hexanal, (E)-2-octenal, 3-methylthiopropionyl aldehyde, decanal, ethyl caproate, 2,5-dimethylpyrazine, and dimethyl trisulfide were the key aroma compounds of DPD, and not all compounds with AI ≥ 2 or OAV ≥ 1 contributed significantly to the omission tests.

### 3.6. Changes in the Contents of Key Aroma Compounds During Storage

**Figure 6:**
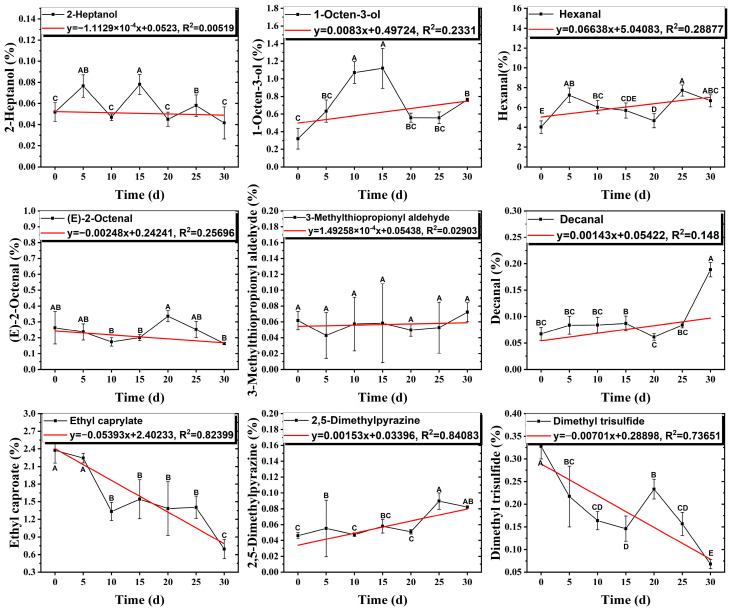
The content of 9 key aroma compounds of DPD in the storage process (25 °C). The red line indicated linear fitting. (A–E) indicate significant differences (*p* < 0.05). Different letters represent significant differences between groups.

**Figure 7:**
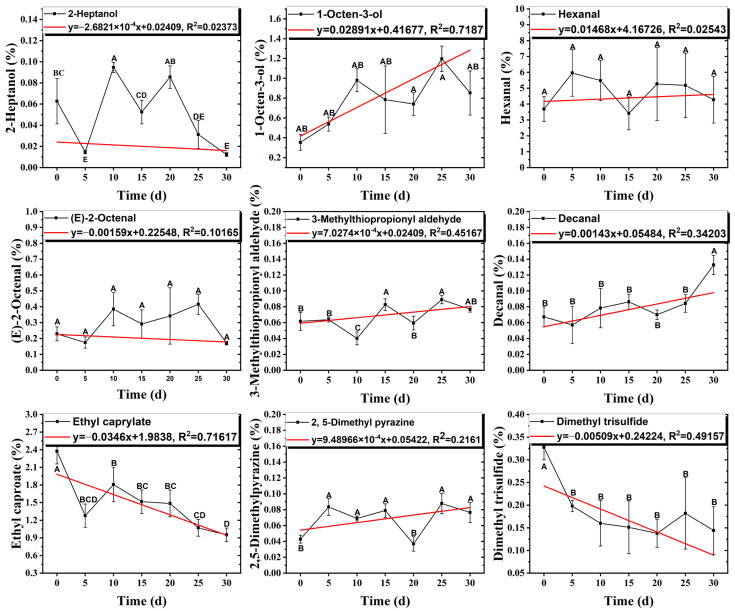
The content of 9 key aroma compounds of DPD in the storage process (4 °C). (A–E) indicate significant differences (*p* < 0.05). Different letters represent significant differences between groups.

Once every five days, the changes in the contents of nine key aroma compounds of DPD samples were detected by GC×GC-MS at different storage temperatures (25 °C, 4 °C) for 30 d. As shown in Figure 6 and Figure 7, during storage, the amounts of aldehydes overall increased, with (E)-2-Octenal showing a slight decreasing trend, which provided greasy and meaty without excessive off-flavors [48]. In addition, the hexanal levels decreased in the middle of storage and then increased, and the hexanal level was the highest on the 25th d at 25 °C. The relative content of hexanal increased with the increase in storage time and the deepening of lipid oxidation, and the high concentration of hexanal showed undesirable odors, such as fatty and rancid flavors [49]. On the 30th d, the decanal content increased steeply, and it might be related to the thermal oxidation of oleic acid [50].

3-Methylthiopropionaldehyde, which was also on the rise, was a sulfur compound produced by the thermal degradation of methionine, but high concentrations of 3-methylthiopropionaldehyde could be unpleasant [34]. During storage, 2-heptanol had a decreasing trend, while the 1-octen-3-ol level tended to increase. 1-Octen-3-ol, derived from lipase-catalyzed reactions and the oxidative decomposition of unsaturated polyunsaturated fatty acids, was an important aroma contributor with a mushroom odor due to its low threshold. Thus, an increase in content could lead to undesirable flavors in the DPD samples [51]. Some esters in DPD might come from spices such as white wine, star anise, and fennel in the formulation, and their low odor thresholds allowed these esters to impart characteristic odors of fruits and flowers [52]. The content of ethyl caproate decreased, and the content of both groups of samples decayed by more than 60%, which affected the characteristic wine flavor of DPD. The content of 2,5-dimethylpyrazine did not change significantly during storage. Given the fact that the threshold of 2,5-dimethylpyrazine was high, it was hypothesized that it did not affect the overall flavor. Dimethyl trisulfide, mainly presenting garlic and onion flavors, was mainly derived from the spices, and its content fluctuated during storage, with the final losses at 25 °C and 4 °C reaching up to 79.3% and 55.9%, respectively. Overall, it could be concluded that the stabilization of key aroma compounds of DPD was more beneficial in storing at 4 °C.

## 4. Conclusions

In this study, five kinds of DPD were systematically analyzed for their key aroma compounds by sensomics approach, and their content changes were then determined by GC×GC-MS during the storage. The results showed that aromas of DPD analyzed by QDA were described as “Meat flavor”, “Grease flavor”, “Garlic flavor”, “Wine flavor”, “Soy Sauce flavor” and “Spice flavor”, and a total of 243 VOCs were identified in the five DPD samples by GC×GC-MS. A total of 16 aroma active compounds, including 1-pentanol, 2-heptanol, 1-octen-3-ol, hexanal, n-octanal, nonanal, (E)-2-octenal, 3-methylthio-propanal, benzaldehyde, decanal, (Z)-2-nonenal, ethyl caproate, 2-pentylfuran, 2,5-dimethylpyrazine, dimethyltrisulfide, and diallyldisulfide, were identified by GC-O-MS and OAV. Finally, based on aroma recombination combined with omission tests, nine compounds, involving 2-heptanol, 1-octen-3-ol, hexanal, (E)-2-octenal, 3-methylthiopropanal, decanal, ethyl caproate, 2,5-dimethylpyrazine, and dimethyl trisulfide, were identified as the key aroma compounds. We monitored the contents of these key aroma compounds at different temperatures (25 °C, 4 °C) during 30 d of storage. Linear fitting revealed that 1-octen-3-ol, hexanal, 3-methylthiopropanal, decanal, and 2,5-dimethylpyrazine showed an increasing trend in their contents during storage, while 2-heptanol, (E)-2-octenal, ethyl hexanoate, and dimethyl trisulfide showed a decreasing trend. Particularly, dimethyl trisulfide (garlicky flavor) and ethyl caproate (wine flavor) showed significant attenuation of more than 50%. The present study systematically investigated the key aroma compounds of DPD as well as their suitable storage conditions. These results would provide scientific guidance for further industrialization production of DPD and its flavor regulation.

## Figures and Tables

**Table 1:** DPD samples information.

Samples	Source Information
A	Laboratory self-made
B	Meizhou Dongpo Restaurant
C	Meizhou Dongpo-Mengnanxuan Restaurant
D	Mingyue Dongpo Restaurant
E	Wangjiadu (Prepared Dishes)

**Table 2:** Aroma description and reference compounds.

Aroma Descriptors	Reference
Meaty	Cooked streaky pork
Greasy	Melted lard
Garlicky	Chopped garlic
Wine flavor	Liquor
Saucy	Mixture of soy sauce and dark soy sauce (1:1)
Spice	Spice powder

**Table 3:** Table of AI and OAV of common compounds of DPD.

	Compounds	Aroma Descriptors	AI					Threshold mg/kg	OAV				
			1	2	3	4	5		1	2	3	4	5
Aldehydes	Hexanal	grass, fatty	3.0	2.0	2.7	2.0	2.0	0.075	2650.7	1967.5	2030.6	1802.8	1669.4
	Octanal	fruity, orange	1.0	2.0	1.5	2.5	0	0.94	31.4	39.2	31.6	23.3	28.1
	Nonanal	rose, oil	2.0	3.3	3.0	2.5	2.0	0.15	351.1	540.1	409.3	288.9	309.4
	(E)-2-Octenal	cucumber, fatty	1.3	2.5	2.0	2.0	0	0.004	2645.5	2710.3	1641.0	2253.5	1166.8
	Methional	onion, broth	3.0	3.3	4.0	2.0	2.5	0.0002	61,838.5	66,480.0	54,595.0	23,730.0	52,270.0
	3-Furaldehyde	-	-	-	-	-	-	-	-	-	-	-	-
	Benzaldehyde	bitter almond	1.7	2.0	3.0	0	2.0	0.06	828.6	1460.3	1705.1	1297.9	1777.4
	Decyl aldehyde	citrus, floral	1.0	3.0	2.5	1.5	2.7	0.65	8.2	16.6	16.7	5.9	12.2
	(Z)-non-2-enal	greasy, rotting grass	-	3.5	4.0	-	-	0.003	1487.9	3601.7	2841.0	1812.7	1984.67
Alcohols	1-Pentanol	fragrance	1.0	3.0	2.5	0	2.0	3	7.2	7.4	4.8	4.8	4.3
	2-Heptanol	lemon, grass	2.7	0	0	0	2.5	0.01	43.1	2297.4	1667.4	1089.2	369.0
	1-Octen-3-ol	mushroom, lavender	2.0	2.0	2.0	2.0	3.0	0.001	26,573.2	34,213.0	29,329.0	21,092.0	29,721.0
	Phenethyl alcohol	floral	1.3	1.0	0	2.0	0	-	-	-	-	-	-
	(S, S)-2,3-Butanediol	-	-	-	-	-	-	-	-	-	-	-	-
Ketones	Acetoin	butter, fatty	1.7	2.0	2.0	2.0	2.0	-	-	-	-	-	-
Ethers	Methyl allyl disulfide	molasses, leek, shallot	1.3	0	0	1.0	0	-	-	-	-	-	-
	Methyl allyl trisulfide	-	-	-	-	-	-	-	-	-	-	-	-
Esters	Ethyl Hexanoate	fruity	1.7	0	3.0	0	0	0.04	1684.9	1160.6	1186.6	428.4	167.1
	Butanoic acid, 2-methyl- 4-methylpentyl ester	-	-	-	-	-	-	-	-	-	-	-	-
Alkanes	Dodecane	fuel	1.3	3.0	0	2.0	0	>13,000	-	-	-	-	-
	1,2,3,4-Tetrathiane, 5-methyl-	-	-	-	-	-	-	-	-	-	-	-	-
Olefins	3-Carene	larch	1.3	2.0	1.0	1.0	2.0	-	-	-	-	-	-
	(1S)- (1)-beta-Pinene	turpentine	-	2.0	1.0	-	-	2.9	3.4	23.6	11.8	5.2	31.9
	(R)-1-methyl-5- (1-methylvinyl) cyclohexene	-	-	-	-	-	-	-	-	-	-	-	-
	Terpinolene	larch, lemon	2.3	2.3	3.0	2.3	0	-	-	-	-	-	-
	1,2-Dithiin, 3-ethenyl-3,6-dihydro-	-	-	-	-	-	-	-	-	-	-	-	-
	β-Caryophyllene	lilac	1.3	0	2	0	1	-	-	-	-	-	-
Pyrazines	2,5-Dimethyl pyrazine	fried peanuts, buttery	2.3	1.0	2.5	2.0	2.0	2.6	15.5	23.0	16.4	13.8	65.6
	2,3-Dimethylpyrazine	buttery, meaty	1.7	2.5	2.5	0	2.0	-	-	-	-	-	-
	2-Ethyl-6-methylpyrazine	nutty, toasty	2.3	0	2.0	2.0	0	-	-	-	-	-	-
Furans	2-Pentylfuran	bean flavor, fruity	1.3	2.0	2.0	1	0	0.1	273.2	435.3	589.7	580.9	1103.2
Sulfides	Dimethyl trisulfide	mint, onion	2.3	2.0	3.0	2.0	0	0.0025	5874.1	7032.4	7220.8	15,257.2	20,192.4
	Diallyldisulfide	molasses	2.0	2.0	2.3	1.0	0	10	30.9	2.7	1.2	0.6	2.8

“-” means not detected or not consulted.

**Table 4:** Standard curve of 16 aroma actives compounds of DPD.

Compounds	CAS	Standard Curve	R^2^
1-Pentanol	71-41-0	y = 0.261x + 117,345	0.994
2-Heptanol	543-49-7	y = 0.595x + 12,010	0.993
1-Octen-3-ol	3391-86-4	y = 0.493x + 16,614	0.990
Hexanal	66-25-1	y = 0.823x + 7473	0.999
n-Octanal	124-13-0	y = 0.644x + 57	0.997
Nonanal	124-19-6	y = 0.547x + 52,873	0.990
(E)-2-Octenal	2548-87-0	y = 0.288x + 60,123	0.999
3-Methylthiopropanal	3268-49-3	y = 0.303x + 535	0.999
Benzaldehyde	100-52-7	y = 0.298x + 20,557	0.990
Decanal	112-31-2	y = 2.082x + 2369	0.998
(Z)-2-Nonenal	60784-31-8	y = 0.032x + 1457	0.992
Ethyl caproate	123-66-0	y = 0.780x + 9992	0.993
2-n-Pentylfuran	3777-69-3	y = 0.037x + 459	0.990
2,5-Dimethylpyrazine	123-32-0	y = 1.179x + 1210	0.998
Dimethyl trisulfide	3658-80-8	y = 0.451x + 2321	0.996
Diallyl disulfide	2179-57-9	y = 0.280x + 1840	0.993

**Table 5:** Influence of aroma compounds on the overall aroma profiles.

Compounds	CAS	RI ^a^	Significance ^b^	n/N ^c^	Chemical Structures
Alcohols			***	12/15	
1-Pentanol	71-41-0	1153	ns	3/15	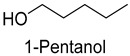
2-Heptanol	543-49-7	1215	**	9/15	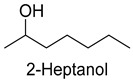
1-Octen-3-ol	3391-86-4	1325	***	12/15	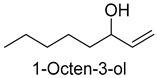
Aldehydes			***	13/15	
Hexanal	66-25-1	979	**	8/15	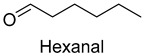
Octanal	124-13-0	1173	ns	7/15	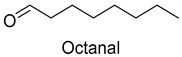
Nonanal	124-19-6	1277	ns	6/15	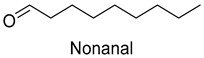
(E)-2-Octenal	2548-87-0	1302	**	9/15	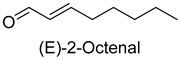
Methional	3268-49-3	1313	**	9/15	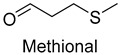
Benzaldehyde	100-52-7	1372	ns	2/15	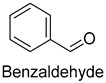
Decanal	112-31-2	1387	*	6/15	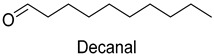
(Z)-2-Nonenal	60784-31-8	1414	ns	5/15	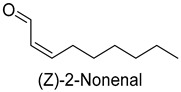
Esters			***	13/15	
Ethyl caproate	123-66-0	1122	***	13/15	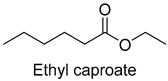
Furans			ns	5/15	
2-Pentylfuran	3777-69-3	1108	ns	5/15	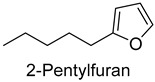
Pyrazines			*	8/15	
2,5-Dimethylpyrazine	123-32-0	1218	*	8/15	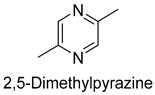
Others			**	10/15	
Dimethyltrisulfide	3658-80-8	1241	**	9/15	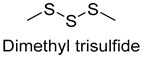
Diallyl disulfide	2179-57-9	1340	ns	3/15	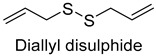

RI ^a^: Retention Index. ^b^ *** very high significance level (*p* ≤ 0.001). **, high significance level (*p* ≤ 0.01). *, significant level (*p* ≤ 0.05). ns, no significant difference (*p* > 0.05). n/N ^c^: Correct number of people/Total number of people. Significance was analyzed by DUNCAN test.

## Data Availability

The data presented in this study are available on request from the corresponding author. The data are not publicly available due to privacy restrictions.

## Supplementary Materials

The following supporting information can be downloaded at: https://www.mdpi.com/article/10.3390/foods14071084/s1, Table S1: Volatile flavor compounds of DPD.

## Abbreviations

The following abbreviations are used in this manuscript:

DPD	Dongpo pork dish
QDA	Quantitative descriptive analysis
GC×GC-MS	Two-dimensional gas chromatography-mass spectrometry
GC-O-MS	Gas chromatography-olfactometry-mass spectrometry
GC-MS	Gas chromatography-mass spectrometry
AI	Aroma intensity
OAV	Odor activity value

## References

[1] Xu J., Zhang M., Wang Y., Bhandari B. (2023). Novel technologies for flavor formation in the processing of meat products: A review. Food Rev. Int..

[2] Fu Y., Cao S., Yang L., Li Z. (2022). Flavor formation based on lipid in meat and meat products: A review. J. Food Biochem..

[3] Tan F., Wang P., Zhan P., Tian H. (2022). Characterization of key aroma compounds in flat peach juice based on gas chromatography-mass spectrometry-olfactometry (GC-MS-O), odor activity value (OAV), aroma recombination, and omission experiments. Food Chem..

[4] Liu H., Hui T., Fang F., Ma Q., Li S., Zhang D., Wang Z. (2021). Characterization and discrimination of key aroma compounds in pre-and postrigor roasted mutton by GC-O-MS, GC E-Nose and aroma recombination experiments. Foods.

[5] De Santis D. (2024). Food Flavor Chemistry and Sensory Evaluation. Foods.

[6] Borràs E., Ferré J., Boqué R., Mestres M., Aceña L., Busto O. (2015). Data fusion methodologies for food and beverage authentication and quality assessment—A review. Anal. Chim. Acta.

[7] Wang S., Chen H., Sun B. (2020). Recent progress in food flavor analysis using gas chromatography–ion mobility spectrometry (GC–IMS). Food Chem..

[8] Liu Z., Phillips J.B. (1991). Comprehensive two-dimensional gas chromatography using an on-column thermal modulator interface. J. Chromatogr. Sci..

[9] Yu M., Yang P., Song H., Guan X. (2022). Research progress in comprehensive two-dimensional gas chromatography-mass spectrometry and its combination with olfactometry systems in the flavor analysis field. J. Food Compos. Anal..

[10] Li W., Zheng L., Xiao Y., Li L., Wang N., Che Z., Wu T. (2022). Insight into the aroma dynamics of Dongpo pork dish throughout the production process using electronic nose and GCŨGC-MS. LWT.

[11] Zhao G., Yuan Y., Zhou H., Zhao L., Jiang Y. (2023). Determination of volatile compounds in different parts of grass carp using GC× GC-MS combined with chemometrics. Food Biosci..

[12] Zhao Y., Zhan P., Geng J., He W., Wang P., Tian H. (2023). Sensomics-assisted key aroma molecules decoding of ginger-infused stewed beef. LWT.

[13] (2017). Technical Specification for Sichuan Cuisine Dongpo Meat Cooking Process.

[14] Wang X., Fan C., Wang X., Feng T., Zhang X., Yu J., Cui H., Xia S. (2023). Microwave heating and conduction heating pork belly: Influence of heat transfer modes on volatile compounds and aroma attributes. Food Biosci..

[15] (2017). Sensory analysis—Methodology—General guidance. Technical Committee ISO/TC 34, Food Products, Subcommittee SC 12, Sensory Analysis.

[16] Zhong A., Chen W., Hu L., Wu Z., Xiao Y., Li K., Li Z., Wang Y., Wang C. (2022). Characterisation of key volatile compounds in fermented sour meat after fungi growth inhibition. LWT.

[17] Nie R., Zhang C., Liu H., Wei X., Gao R., Shi H., Zhang D., Wang Z. (2024). Characterization of key aroma compounds in roasted chicken using SPME, SAFE, GC-O, GC–MS, AEDA, OAV, recombination-omission tests, and sensory evaluation. Food Chem. X.

[18] Jiang K., Xu K., Wang J., Meng F., Wang B. (2023). Based on HS-SPME-GC-MS combined with GC-O-MS to analyze the changes of aroma compounds in the aging process of Citri Reticulatae Pericarpium. Food Biosci..

[19] Wang L., Wu L., Xiang D., Huang H., Han Y., Zhen P., Shi B., Chen S., Xu Y. (2023). Characterization of key aroma compounds in aged Qingxiangxing baijiu by comparative aroma extract dilution analysis, quantitative measurements, aroma recombination, and omission studies. Food Chem..

[20] Zhang Z., He S., Zhang L., Li X., Jin R., Liu Q., Chen S., Wang J., Sun H. (2022). The potential application of vegetable oils in the D-xylose and L-cysteine Maillard reaction system for meaty aroma production. Food Res. Int..

[21] Yang X., Liu J., Wan P., Guo D., Chen D.-W. (2022). Use of egg yolk to imitate meat aroma. Food Chem..

[22] Zhang L., Hu Y., Wang Y., Kong B., Chen Q. (2021). Evaluation of the flavour properties of cooked chicken drumsticks as affected by sugar smoking times using an electronic nose, electronic tongue, and HS-SPME/GC-MS. LWT.

[23] Wang S., Chen H., Sun J., Zhang N., Wang S., Sun B. (2023). Effects of cooking methods on aroma formation in pork: A comprehensive review. Food Chem. X.

[24] Hoa V.-B., Seol K.-H., Seo H.-W., Kang S., Kim Y., Seong P., Moon S., Kim J., Cho S. (2021). Investigation of physicochemical and sensory quality differences in pork belly and shoulder butt cuts with different quality grades. Food Sci. Anim. Resour..

[25] Cheng L., Li X., Tian Y., Wang Q., Li X., An F., Luo Z., Shang P., Liu Z., Huang Q. (2023). Mechanisms of cooking methods on flavor formation of Tibetan pork. Food Chem. X.

[26] Ahamed Z., Seo J.-K., Eom J.-U., Yang H.-S. (2023). Optimization of volatile compound extraction on cooked meat using HS-SPME-GC-MS, and evaluation of diagnosis to meat species using volatile compound by multivariate data analysis. LWT.

[27] Zhang M., Zhou C., Zhang C., Xu K., Lu L., Huang L., Zhang L., Li H., Zhu X., Lai Z. (2023). Analysis of Characteristics in the Macro-Composition and Volatile Compounds of Understory Xiaobai White Tea. Plants.

[28] Wu T., Wang P., Zhang Y., Zhan P., Zhao Y., Tian H., He W. (2023). Identification of muttony-related compounds in cooked mutton tallows and their flavor intensities subjected to phenolic extract from thyme (*Thymus vulgaris* L.). Food Chem..

[29] He Z.-G., Zhang Y., Yang M.-D., Zhang Y.-Q., Cui Y.-Y., Du M.-Y., Zhao D., Sun H. (2022). Effect of different sweeteners on the quality, fatty acid and volatile flavor compounds of braised pork. Front. Nutr..

[30] Gąsior R., Wojtycza K., Majcher M.A., Bielińska H., Odrzywolska A., Bączkowicz M., Migdał W. (2021). Key aroma compounds in roasted White Kołuda Goose. J. Agric. Food Chem..

[31] Chang Y., Wang S., Chen H., Zhang N., Sun J. (2021). Characterization of the key aroma compounds in pork broth by sensory-directed flavor analysis. J. Food Sci..

[32] Zhang J., Zhong L., Wang P., Song J., Shi C., Li Y., Oyom W., Zhang H., Zhu Y., Wen P. (2024). HS-SPME-GC-MS Combined with Orthogonal Partial Least Squares Identification to Analyze the Effect of LPL on Yak Milk’s Flavor under Different Storage Temperatures and Times. Foods.

[33] Lyte J.M., Legako J.F., Martin J.N., Thompson L., Surowiec K., Brooks J. (2016). Volatile compound characterization of modified atmosphere packaged ground beef held under temperature abuse. Food Control.

[34] Bi J., Li Y., Yang Z., Lin Z., Chen F., Liu S., Li C. (2022). Effect of different cooking times on the fat flavor compounds of pork belly. J. Food Biochem..

[35] Wang Y., Zhang H., Cui J., Gao S., Bai S., You L., Ji C., Wang S. (2024). Dynamic changes in the water and volatile compounds of chicken breast during the frying process. Food Res. Int..

[36] Qiu D., Gan R., Feng Q., Shang W., He Y., Li C., Shen X., Li Y. (2024). Flavor formation of tilapia byproduct hydrolysates in Maillard reaction. J. Food Sci..

[37] Yao W., Cai Y., Liu D., Chen Y., Li J., Zhang M., Chen N., Zhang H. (2022). Analysis of flavor formation during production of Dezhou braised chicken using headspace-gas chromatography-ion mobility spec-trometry (HS-GC-IMS). Food Chem..

[38] Wen R., Sun F., Li X.-A., Chen Q., Kong B. (2021). The potential correlations between the fungal communities and volatile compounds of traditional dry sausages from Northeast China. Food Microbiol..

[39] Jiang F., Zhang J., Zhang R., Zhang W. (2024). Effects of ultrasound-assisted vacuum tumbling on the flavor of spiced beef. Food Biosci..

[40] Hu G., Cai K., Li Y., Hui T., Wang Z., Chen C., Xu B., Zhang D. (2021). Significant inhibition of garlic essential oil on benzo [a] pyrene formation in charcoal-grilled pork sausages relates to sulfide compounds. Food Res. Int..

[41] Shi Y., Chen G., Chen K., Chen X., Hong Q., Kan J. (2021). Assessment of fresh star anise (Illicium verum Hook. f.) drying methods for influencing drying characteristics, color, flavor, volatile oil and shikimic acid. Food Chem..

[42] Gopinath A., Yadav S.A., Ranjithkumar D. (2023). Evaluation of Comparative metabolomic profile in Cardamom elettaria and Amomum subulatum fruits. Orient. J. Chem..

[43] Gooderham N.J., Cohen S.M., Eisenbrand G., Fukushima S., Guengerich F.P., Hecht S.S., Rietjens I.M., Rosol T.J., Davidsen J.M., Harman C.L. (2020). FEMA GRAS assessment of natural flavor complexes: Clove, cinnamon leaf and West Indian bay leaf-derived flavoring ingredients. Food Chem. Toxicol..

[44] Kunihiro K., Kikuchi Y., Nojima S., Myoda T. (2022). Characteristic of aroma components and antioxidant activity of essential oil from Ocimum tenuiflorum leaves. Flavour Fragr. J..

[45] Jiang H., Zhang M., Ye J., Qian M., Li X., Zhao W., Bai W. (2022). HS-SPME-GC-MS and OAV analyses of characteristic volatile flavour compounds in salt-baked drumstick. LWT.

[46] Yang X., Pei Z., Du W., Xie J. (2023). Characterization of Volatile Flavor Compounds in Dry-Rendered Beef Fat by Different Solvent-Assisted Flavor Evaporation (SAFE) Combined with GC–MS, GC–O, and OAV. Foods.

[47] Zhao Q., Shen Q., Guo R., Wu J., Dai Z.-Y. (2016). Characterization of flavor properties from fish (Collichthys niveatus) through enzymatic hydrolysis and the maillard reaction. J. Aquat. Food Prod. Technol..

[48] Cui H., Liu D., Xia X., Yu J., Hayat K., Zhang X., Ho C.-T. (2021). Flavor and texture characteristics of microwave-cooked Kung Pao Chicken by different heat conduction effects and further aroma improvement with moderate enzymatic hydrolyzed chicken fat. Food Funct..

[49] Duan Z., Dong S., Sun Y., Dong Y., Gao Q. (2021). Response of Atlantic salmon (Salmo salar) flavor to environmental salinity while culturing between freshwater and seawater. Aquaculture.

[50] Hoa V.-B., Song D.-H., Min Y.-J., Seol K.-H., Kang S.-M., Kim H.-W., Moon S.-S., Cho S.-H. (2023). Carcass trait, meat yield and quality characteristics of recently-synthesized Woori Heukdon and commercial LYD pigs under identical rearing condition. Anim. Biosci..

[51] Zhang K., Zang M., Wang S., Zhang Z., Li D., Li X., Zhao Y. (2023). Effects of defatting and cooking methods on pig large intestines volatile compounds by flavor omics and fatty acids. LWT.

[52] Liu H., Wang Z., Zhang D., Shen Q., Pan T., Hui T., Ma J. (2019). Characterization of key aroma compounds in Beijing roasted duck by gas chromatography–olfactometry–mass spectrometry, odor-activity values, and aroma-recombination experiments. J. Agric. Food Chem..

