# Supplementary material for: Characterization of Key Aroma Compounds in Dongpo Pork Dish and Their Dynamic Changes During Storage
Source: Foods. 2025 Mar 21;14(7):1084. doi: 10.3390/foods14071084 (PMC11988299; doi:10.3390/foods14071084)
Supplement: Supplementary file 1 [file foods-14-01084-s001.zip › foods-3495638-supplementary.pdf]

Supplementary Materials

Table S1. Volatile flavor compounds of DPD

Compounds	CAS	RI	Concentration mg/kg				
			1	2	3	4	5
Hexanal	66-25-1	979	1988.006	147.563	152.298	135.213	125.202
Octanal	124-13-0	1173	294.815	36.879	29.683	21.897	26.384
(Z)-2-Heptenal	57266-86-1	1207	161.628	24.074	20.182	-	-
Nonanal	124-19-6	1277	526.604	81.021	61.39	43.345	46.41
(E)-Oct-2-enal	2548-87-0	1305	105.818	10.841	6.564	9.014	4.667
3-Methylthiopropanal	3268-49-3	1313	123.677	13.296	10.919	4.746	10.454
3-Furaldehyde	498-60-2	1316	51.132	14.249	16.13	11.088	9.924
Benzaldehyde	100-52-7	1372	497.163	87.62	102.305	77.876	106.646
Decanal	112-31-2	1387	53.096	10.771	10.871	3.848	7.953
(Z)-2-Nonen-1-al	60784-31-8	1414	44.638	10.805	8.523	5.438	5.954
Phenylacetaldehyde	122-78-1	1480	226.942	-	-	34.248	-
Anisic aldehyde	123-11-5	1824	143.39	16.153	19.206	16.433	-
(2E)-2-Decenal	3913-81-3	1515	-	4.87	-	3.372	-
(Z)-Citral	106-26-3	1541	-	17.218	11.608	16.012	-
(E)-Citral	141-27-5	1586	-	11.355	11.191	-	-
Lauryl aldehyde	112-54-9	1589	-	0.724	0.604	-	-
(Z)-Cinnamaldehyde	57194-69-1	1839	-	5.267	5.789	13.166	-
1-Hexadecanal	629-80-1	1991	-	4.071	8.901	9.757	-
(−)-Myrtenal	18486-69-6	1490	-	-	2.849	1.181	-
Piperonal	120-57-0	1998	-	-	0.341	0.51	0.563
Z-9-Octadecenal	2423-10-1	2203	-	-	0.389	0.62	-

1H-Indene-4-carboxaldehyde, 2,3-dihydro-(9CI)	51932-70-8	1795	-	-	0.612	0.922	-
(E)-3-Phenylbut-2-enal	1196-67-4	1850	-	-	0.341	-	-
Undecenal	2463-77-6	1615	-	-	-	1.639	-
2-Phenyl-2-butenal	4411-89-6	1747	-	-	-	0.56	1.036
Phenylpropyl aldehyde	104-53-0	1608	-	-	-	5.199	-
(E, E)-2,4-Dodecadienal	21662-16-8	1615	-	-	-	2.764	-
5-Methyl-2-furaldehyde	620-02-0	1420	-	-	-	-	18.23
Cuminaldehyde	122-03-2	1622	-	-	-	-	6.112
4-Propylbenzaldehyde	28785-06-0	1662	-	-	-	-	17.158
Octadecanaldehyde	638-66-4	1991	-	-	-	-	6.961
5-Methylhexanal	1860-39-5	1075	-	-	-	-	44.633
Cineole	470-82-6	1108	110.644	211.241	206.39	201.693	-
Pentan-1-ol	71-41-0	1118	215.745	22.156	14.419	14.286	12.873
Methyl (1E)-1-propen-1-yl	23838-19-9	1153	314.75	1.053	-	-	-
2-Heptanol	543-49-7	1215	4.307	22.974	16.674	10.892	3.69
1-Hexanol	111-27-3	1243	65.562	-	-	-	-
Oct-1-en-3-ol	3391-86-4	1325	265.732	34.213	29.329	21.092	29.721
(2R,3R)-(-)-2,3-Butanediol	24347-58-8	1398	128.837	-	-	-	-
Linalool	78-70-6	1420	110.111	22.567	21.433	13.311	-
Terpinine-4-ol	562-74-3	1471	150.019	8.223	7.6	6.134	-
3H-1,2-Dithiole	118023-96-4	1563	62.311	-	-	-	-
Phenethyl alcohol	60-12-8	1722	56.494	10.352	8.781	5.298	10.868
(2S,3S)-(+)-2,3-BUTANEDIOL	19132-06-0	1438	158.7	24.467	8.913	8.764	45.464
(+)-alpha-terpineol	7785-53-7	1556	138.364	-	-	3.372	-
Ethanol	64-17-5	847	-	2.79	5.073	-	-

4-Methyl-1-pentanol	626-89-1	1210	-	24.331	19.411	9.543	35.834
2-Methylheptan-3-ol	18720-62-2	1229	-	0.819	1.56	-	-
2-Nonanol	628-99-9	1399	-	9.272	7.443	4.977	-
(±)-Neoisopulegol	29141-10-4	1444	-	1.203	-	-	-
2-Methyloctan-1-ol	818-81-5	1459	-	20.008	6.189	-	-
(E)-Oct-2-en-1-ol	18409-17-1	1474	-	25.375	32.248	6.608	11.393
1-Nonanol	143-08-8	1483	-	42.306	63.536	-	-
Furfuryl alcohol	98-00-0	1495	-	40.872	68.454	30.403	34.29
5-Methyl-2-furanmethanol	3857-25-8	1553	-	5.305	5.279	3.374	8.204
3-Methyl-1-butanol	123-51-3	1124	-	10.249	13.436	9.862	25.551
Bicyclo[2.2.1]heptan-2-ol, 2,7,7-trimethyl-, endo-	3247-40-3	1352	-	8.686	-	16.337	-
2,3-Dimethylcyclohexanol	1502-24-5	1357	-	5.07	-	7.408	-
Cyclohexanol, 2,4-dimethyl-	69542-91-2	1408	-	7.042	11.061	6.89	9.916
Adamantan-1-ol	768-95-6	1173	-	-	0.892	-	-
2-Ethylhexan-1-ol	104-76-7	1363	-	-	5.275	-	27.487
(6S)-6-Methyl-1-octanol	110453-78-6	1432	-	-	34.447	-	-
(2S,4R)-(-)-2,4-dimethylheptan-1-ol	18450-74-3	1450	-	-	28.349	-	-
1-Hexadecanol	36653-82-4	1998	-	-	2.63	-	-
(±)-Myrtenol	515-00-4	1632	-	-	2.838	2.198	-
Benzyl alcohol	100-51-6	1688	-	-	2.508	2.108	5.679
Thiophen-2-ylmethanol	636-72-6	1743	-	-	2.324	-	-
(Z)-Hex-3-en-1-ol	928-96-1	1268	-	-	-	0.72	1.718
2-Ethyl-4-methyl-1-pentanol	106-67-2	1363	-	-	-	2.991	-
1-Octanol,2-butyl-	3913-02-8	1151	-	-	-	-	2.898
2-ethylfenchol	67952-68-5	1450	-	-	-	-	8.915

Orcinol	504-15-4	1459	-	-	-	-	6.008
Alpha-Terpineol	10482-56-1	1553	-	-	-	-	13.054
3-Hexanone,2,5-dimethyl-	1888-57-9	1063	503.025	-	32.288	34.693	-
Acetoin	513-86-0	1184	57.072	12.445	8.609	5.983	9.275
Acetol	116-09-6	1193	42.068	-	-	6.911	13.311
6-Methylhept-5-en-2-one	110-93-0	1218	13.326	58.322	58.634	45.565	-
Fenchone	1195-79-5	1277	99.595	-	-	-	-
Piperitone	89-81-6	1586	163.739	-	-	-	-
Gamma-chloro-2-butyrothienone	43076-59-1	1602	70.072	-	-	-	-
9-Oxabicyclo[3.3.1]nonan-2-one	35519-67-6	1648	106.429	-	-	-	18.022
4',6'-Dimethoxy-2'-hydroxyacetophenone	90-24-4	2334	34.406	-	-	-	-
4-Octanone	589-63-9	1062	4268.836	-	-	-	-
2,3-Pentanedione	600-14-6	949	-	8.692	6.016	-	6.513
2-Methyltetrahydrofuran-3-one	3188-00-9	1147	-	5.299	5.68	3.718	10.784
Methyl nonyl ketone	112-12-9	1484	-	6.842	4.547	2.312	3.325
2-Cyclohexen-1-one,4-(1-methylethyl)-	500-02-7	1528	-	7.299	5.35	3.533	1.073
2,6,6-trimethylcyclohepta-2,4-dien-1-one	503-93-5	1553	-	3.12	2.991	2.33	0.745
2-Tridecanone	593-08-8	1682	-	2.443	1.989	1.259	2.6
4H-2,3b-Methanocyclopropa[1,2:1,3]dicyclopenten-3(3aH)-one	16492-06-1	1702	-	1.501	-	1.155	-
4-Hydroxy-2,5-dimethylfuran-3(2H)-one	3658-77-3	1824	-	4.687	3.305	-	6.532
2-Pentadecanone	2345-28-0	1880	-	6.987	5.883	3.664	7.633
5-Methyl-2-hexanone	110-12-3	1074	-	21.391	-	-	-
4H-pyran-4-one, 2,3-dihydro-3,5-dihydroxy-6-methyl	28564-83-2	2022	-	1.494	-	-	-
2-Heptanone	110-43-0	1075	-	-	65.167	-	-

4-Methoxyphenylacetone	122-84-9	1944	-	-	0.668	0.826	-
1,3-Dithiole-2-thione	930-35-8	2223	-	-	4.256	-	15.96
3-Methylbutyrolactone	1679-49-8	1456	-	-	-	1.096	-
(E)-6-Methyl-3,5-heptadien-2-one	16647-04-4	1456	-	-	-	3.63	-
2'-Hydroxy-5'-methylpropiophenone	938-45-4	2098	-	-	-	0.515	-
4-Methylheptan-2-one	6137-06-0	1102	-	-	-	-	4.105
3-Heptanone,2,6-dimethyl	19549-83-8	1164	-	-	-	-	13.147
10-Methylicosane	54833-23-7	1332	-	-	-	-	1.964
3-Butyloxolan-2-one	19340-56-8	1465	-	-	-	-	12.332
2-Acetyl-6-methyl pyrazine	22047-26-3	1540	-	-	-	-	7.729
Oxacyclononadec-10-en-2-one	80060-76-0	2372	-	-	-	-	3.562
2-Cyclopentylcyclopentanone	4884-24-6	2110	-	-	-	-	1.059
4-Methylpentyl isobutyrate	35852-44-9	1193	62.6	7.587	3.121	2.727	-
Octanoic acid	124-07-2	1868	-	22.286	19.372	15	-
Decanoic acid	334-48-5	2066	-	3.512	2.404	-	9.215
Hexadecanoic acid-1-13C	1957-10-3	2290	-	8.709	-	-	-
(Z)-13-Octadecenoic acid	693-71-0	2421	-	25.892	-	-	-
Cis-8,11,14-Eicosatrienoic acid	1783-84-2	2335	-	7.415	-	-	-
3-Methylbutanoic acid	503-74-2	1514	-	-	-	4.607	-
1-Hexanoic acid	142-62-1	1671	-	-	-	-	15.138
Butylated hydroxytoluene	128-37-0	1757	79.999	-	-	1.148	-
Methyleugenol	93-15-2	1828	40.615	-	-	-	-
Phenol, 2-methoxy-3-(2-propenyl)	1941-12-4	1948	222.268	-	-	-	-
2,5-Di-tert-butylphenol	5875-45-6	2090	15.26	-	-	-	0.61
isoeugenol	97-54-1	2106	22.862	0.398	0.59	0.865	-
Phenol	108-95-2	1795	-	5.116	6.689	5.475	6.027

4-Hydroxy-3-methoxystyrene	7786-61-0	1967	-	5.284	4.318	1.834	1.04
2,5-diethyl phenol	876-20-0	1990	-	1.575	-	-	-
Mequinol	150-76-5	1671	-	4.526	-	3.995	14.15
Creosol	93-51-6	1760	-	-	1.461	0.865	0.952
2-Methoxy-5-prop-2-enyl-phenol	501-19-9	1948	-	-	0.661	0.637	-
3-Ethyl-5-methylphenol	698-71-5	1990	-	-	1.774	-	-
5-Methyl-2-prop-1-en-2-ylphenol	18612-99-2	1795	-	-	0.893	0.952	-
m-Cresol	108-39-4	1872	-	-	-	-	20.751
5-Isopropyl-2-methylphenol	499-75-2	1967	-	-	-	-	5.046
Phenol,2-ethyl-4,5-dimethyl-	2219-78-5	1990	-	-	-	-	1.674
Ethyl valerate	539-82-2	1032	104.946	-	-	-	-
Ethyl hexanoate	123-66-0	1119	673.961	46.424	47.463	17.136	6.685
Butanoic acid, 2-methyl-, 4-methylpentyl ester	35852-40-5	1280	83.878	12.302	6.661	5.207	27.817
Ethyl 2-Hydroxybutyrate	52089-54-0	1282	34.631	-	-	-	-
Ethyl 2-hydroxy-3-methyl butyrate	2441-06-7	1299	105.44	-	-	-	-
12,15-Octadecadiynoic acid methyl ester	57156-95-3	1020	71.945	-	7.349	-	-
4-Methyl pentyl isovalerate	850309-45-4	1296	96.129	14.115	6.778	-	29.313
3-Buten-1-yl isothiocyanate	3386-97-8	1313	-	4.019	-	5.622	-
Exyl 2-methylbutanoate	10032-15-2	1323	-	1.081	-	-	3.578
Cyclopropanecarboxylic acid, decyl ester	54460-47-8	1489	-	51.727	66.554	-	-
Isobornyl formate	1200-67-5	1553	-	27.95	27.176	19.783	-
α-Terpinyl acetate	80-26-2	1563	-	0.666	0.589	1.065	-
Dodecyl formate	28303-42-6	1806	-	1.821	-	0.622	0.876
Diethyl adipate	141-28-6	1733	-	15.473	11.285	-	-

Ethyl cinnamate	4192-77-2	1929	-	1.684	1.012	1.07	-
δ-Decalactone	705-86-2	1991	-	2.096	1.961	1.201	0.991
4-METHYLPENTYL 4-METHYLVALERATE	35852-42-7	1423	-	-	1.14	-	3.439
Decyl formate	5451-52-5	1524	-	-	10.915	-	-
[(1R,2S,5R)-5-Methyl-2-prop-1-en-2-ylcyclohexyl] acetate	57576-09-7	1540	-	-	5.018	-	-
Heptyl formate	112-23-2	1334	-	-	-	4.36	-
1-α-Linolenoylglycerol	18465-99-1	2494	-	-	-	3.217	-
Butanoic acid, 3-methyl-, 4,6,6-trimethylbicyclo[3.1.1]hept-3-en-2-yl ester, (1α,2α,5α)- (9CI)	57412-35-8	1060	-	-	-	-	10.589
Butanoic acid,2-methyl-, 3-methylbutyl ester	27625-35-0	1179	-	-	-	-	4.07
Hexyl isobutyrate	2349-7-7	1199	-	-	-	-	17.044
Ethyl caprate	110-38-3	1528	-	-	-	-	1.121
Methyl salicylate	119-36-8	1608	-	-	-	-	8.655
Ethyl 4-methyl valerate	25415-67-2	1081	-	-	-	-	6.531
2'-Hexyl-1,1'-bicyclopropane-2-octanoic acid methyl ester	56687-68-4	1675	-	-	-	-	12.582
Allyl Sulfide	592-88-1	1026	1305.123	9.039	-	-	33.305
Methyl allyl disulfide	2179-58-0	1145	1354.25	29.108	19.481	17.274	39.858
Allyl Propyl Sulfide	27817-67-0	1307	71.057	-	-	-	-
3H-1,2-Dithiole	288-26-6	1366	375.021	5.248	-	-	10.865
Methyl allyl trisulfide	34135-85-8	1438	676.894	14.504	7.819	8.593	21.579
Diallyl trisulfide	2050-87-5	1629	126.293	-	-	-	3.018
Estragole	140-67-0	1662	2211.479	107.805	91.273	95.894	-
Myristicin	607-91-0	2042	16.413	-	-	-	-
γ-Asarone	5353-15-1	2018	12.005	-	-	-	-

2-Furfuryl methyl sulfide	1438-91-1	1343	-	-	-	0.431	-
allyl 1-propenyl disulfide	122156-02-9	1346	1193.72	11.174	2.096	-	-
Dodecane	112-40-3	1131	26.992	27.085	19.602	11.457	29.674
2-Ethylidene-1,3-dithiane	51102-62-6	1534	68.738	-	-	-	-
5-Methyltetrathiane	116664-30-3	1836	165.708	2.906	3.22	3.229	30.508
Disulfide, 1-methylethyl 2-propen-1-yl	67421-85-6	1299	27.097	-	-	-	-
1-Cyclopropylpentane	2511-91-3	1429	116.65	-	-	8.224	-
Decane	124-18-5	953	-	66.95	-	-	26.34
(-)-CAMPHENE	5794-04-7	976	-	128.907	61.711	50.175	-
n-Hendecane	1120-21-4	996	-	61.418	22.249	33.789	43.525
N-PENTADECANE	629-62-9	1194	-	19.969	4.599	5.179	2.513
N-HEXADECANE-D34	544-76-3	1238	-	7.64	3.049	-	12.301
Tetradecane	629-59-4	1361	-	3.971	5.236	3.397	7.326
2,6,10-trimethyltetradecane	14905-56-7	1423	-	2.981	-	-	-
1-(1,5-Dimethyl-4-hexenyl)-4-methylenebicyclo[3.1.0]hexane	58319-04-3	1652	-	1.518	-	-	-
Undecane, 3-methylene-	71138-64-2	1182	-	3.386	-	3.613	7.721
2,6,11-TRIMETHYLDODECANE	31295-56-4	1230	-	-	1.65	-	-
3-METHYLTRIDECANE	6418-41-3	1305	-	-	2.842	-	4.461
Cyclene	508-32-7	1042	-	-	-	16.276	-
3-Methylundecane	1002-43-3	1105	-	-	-	-	13.252
1,2,3-Trithiolane, 4-methyl-	116664-29-0	1592	-	-	-	-	27.576
Carene	13466-78-9	1045	234.592	232.794	123.47	60.503	477.937
(1S)-(1)-beta-Pinene	18172-67-3	1012	99.792	68.575	34.09	15.039	92.738

(R)-1-methyl-5-(1-methylvinyl)cyclohexene	1461-27-4	1081	1956.951	158.993	138.866	55.881	298.543
Terpinolene	586-62-9	1159	22.557	10.671	2.702	3.236	13.8
(-)-ALPHA-COPAENE	3856-25-5	1414	7.373	1.212	-	-	1.528
1-Octene, 3,7-dimethyl-	4984-1-4	1429	56.818	-	-	-	18.366
Isocaryophyllene	118-65-0	1478	7.537	-	2.331	-	4.904
3-Ethenyl-3,6-dihydrodithiine	62488-52-2	1573	757.993	24.327	16.775	10.664	38.4
Alpha-Curcumene	644-30-4	1646	12.146	8.687	3.511	2.984	-
ALPHA, P-DIMETHYLSTYRENE	1195-32-0	1319	67.256	-	-	-	-
β-Caryophyllene	87-44-5	1496	11.225	11.904	3.859	5.042	17.04
Alpha-pinene	80-56-8	945	-	85.011	27.416	17.925	71.775
(+)-Camphene	79-92-5	988	-	53.395	-	-	-
β-Phellandrene	555-10-2	1090	-	87.797	47.569	70.298	-
Styrene	100-42-5	1125	-	4.75	7.287	6.129	-
Para-menthatriene	18368-95-1	1269	-	0.723	-	-	2.691
Zingiberene	495-60-3	1606	-	3.535	-	-	-
Gamma-muurolene	30021-74-0	1609	-	1.797	-	-	-
Caryophyllene	13877-93-5	1478	-	2.288	0.918	-	-
(1S,3S)-(E)-4-carene	5208-50-4	1119	-	-	2.412	-	-
β-Bisabolene	495-61-4	1616	-	-	0.497	-	-
β-Sesquiphellandrene	20307-83-9	1652	-	-	0.515	-	-
1,5,5-Trimethyl-6-methylenecyclohexene	514-95-4	1033	-	-	-	4.707	14.143
Alpha-Terpinene	99-86-5	1069	-	-	-	-	3.668
Alpha-caryophyllene	6753-98-6	1560	-	-	-	-	1.631
2-Amylfuran	3777-69-3	1105	273.21	43.526	58.966	58.085	110.324
(2S,3S)-2-ethyl-3-methylthiolane	61568-37-4	1271	34.151	-	-	-	-

3-Methyl-1-benzofuran	21535-97-7	1843	74.491	-	-	-	-
3-Methyl-4,5-dihydrofuran	34314-83-5	1093	-	-	5.037	-	-
1-(Furan-2-yl)ethanone	1192-62-7	1357	-	-	10.531	-	16.757
2,5-Dimethylpyrazine	123-32-0	1218	402.805	59.834	42.623	35.969	170.684
2,3-Dimethylpyrazine	5910-89-4	1238	56.396	6.936	5.945	4.41	11.522
Pyrazine,2-ethyl-6-methyl-	13925-03-6	1271	139.484	9.222	7.332	5.185	30.803
2,3,5-Trimethylpyrazine	14667-55-1	1293	-	17.328	17.853	20.353	44.644
2-Ethyl-3,5-dimethylpyrazine	13360-65-1	1331	-	-	5.983	5.456	19.281
2,6-Dimethylpyrazine	108-50-9	1218	-	-	-	20.392	-
Tetramethylpyrazine	1124-11-4	1361	-	-	-	4.236	30.219
2-METHYL-6-VINYLPYRAZINE	13925-09-2	1357	-	-	-	-	11.78
Dimethyl trisulfide	3658-80-8	1238	146.853	17.581	18.052	38.143	50.481
Diallyl disulfide	2179-57-9	1340	3085.494	26.871	11.83	5.978	27.884
o-Cymene	527-84-4	1145	351.606	108.853	74.35	38.843	122.125
2-Vinylthiophene	1918-82-7	1162	36.453	-	-	-	-
2-Methylpyrimidine	5053-43-0	1161	79.065	17.994	17.474	12.275	34.103
Delta-elemene	20307-84-0	1373	-	5.34	-	-	13.483
(±)-Camphor	464-48-2	1384	-	1.315	-	-	-
2H-Cyclopenta[b]thiophene, hexahydro-, trans- (9CI)	53956-12-0	1648	-	9.733	14.708	-	-
Acetic anhydride	108-24-7	1204	-	10.43	11.042	-	-
6-Methylindole	3420-2-8	2214	-	-	1.373	-	-
P-XYLENE	106-42-3	1026	-	-	-	10.777	-
2H-Cyclopenta[b]thiophene, hexahydro-, cis- (9CI)	53956-11-9	1648	-	-	-	9.045	-
4,5-Dimethylthiazole	3581-91-7	1254	-	-	-	-	1.009

4,7-Ethanobenzo[c]thiophene, octahydro-, cis- (9CI)	54411-18-6	1438	-	-	-	-	8.636
3-(4-Methyl-3-pentenyl) thiophene	62429-57-6	1502	-	-	-	-	2.173
2-Acetyl-2-thiazoline	29926-41-8	1586					1.983

“RI” means Retention Index；“-” means not detected.
